# Childhood Diarrhoea: Failing Conventional Measures, what Next?

**DOI:** 10.4314/pamj.v8i1.71164

**Published:** 2011-04-24

**Authors:** Faraj Alkizim, Duncan Matheka, Anne Muriithi

**Affiliations:** 1 Department of Medical Physiology, University of Nairobi

**Keywords:** Diarrhoea research, faecal biotherapy, Gastroenteritis, Infant mortality, MDG, Nonconventional treatment

## Abstract

**Background:**

Diarrhoea is one of the leading causes of infant mortality. This article analyzes its contribution towards the realization of millennium development goal number 4 (MDG-4).

**Methods:**

A PubMed search using keywords acute infant diarrhea together with prevalence, management, or prevention 23 of the 634 generated articles were reviewed for inclusion.

**Results:**

WHO first expressed concern about diarrhoeal mortality in 1979. Two decades later it reported diarrhoea as the second leading cause of infant mortality worldwide. The annual death toll of 1.5 million is greater than AIDS, malaria and measles combined. Short term repercussions (dehydration, electrolyte imbalance, malnutrition, shock, death) plus long-term diminished fitness index, cognitive function, and school performance have major impact on society. Ever since its 1971 success, Oral Rehydration Therapy has been the cornerstone treatment of diarrhoea. Decreased compliance has been recorded worldwide with Kenya ranking first. Intravenous therapy is useful in preventing complications while anti-diarrhoeals and anti-microbials, are indicated in severe cases. Zinc supplementation has also proven effective, and is recommended along with rehydration. Furthermore, immunization and good hygiene prevent faecal-oral transmissions.

**Conclusion:**

MDG-4 aims to reduce childhood mortality by 2/3 by 2015. Studies, however, show minimal progress, and the target is likely to be missed. Efforts must therefore be made to review existing strategies and formulate newer ones. Research priorities need to move away from perceived ‘killer diseases’ since far more children die in a day than have ever died from avian influenza for example.

## Background

Diarrhoea, as defined by the World Health Organization (WHO), is the passage of loose stool by an individual, at least three times a day, or more frequently than normal [1]. It usually lasts less than 7 days; when it lasts longer than 14 days, it is called “protracted diarrhoea” [2]. It is most commonly caused by intestinal infection [3], mainly viral [2], that is transmitted faecal-orally.

The main causes of concern are the loss of body water and electrolyte imbalance. Children are also at greater risk than adults of life-threatening dehydration since water constitutes a greater proportion of their bodyweight; in fact, the younger the child, the greater the risk of dehydration. The type of dehydration (isotonic, hypotonic, or hypertonic) is independent of the causative organism [2]. Fluid losses resulting from diarrhoea and vomiting can be as high as three times the circulating blood volume. In order to keep intravascular volume constant, water is lost from the intracellular compartment to the extracellular, leading to dehydration [2].

With repeated attacks, protein energy malnutrition results, and if not contained, complications such as renal failure and subsequently death will occur. Malnutrition on the other hand, is a predisposing factor to the increased frequency and severity of diarrhoea. The disease is, therefore, closely related to protein energy malnutrition [4]; this is known as the “vicious cycle of diarrhoea” [5], a cycle that could consequently lead to impaired growth and development [3].

Studies on the long-term effects of childhood diarrhoea have also been performed. Decreased physical fitness, declined cognitive function, delayed school commencement, and poor school performance have all been shown to be repercussions of early childhood diarrhoea [5]. This makes the disease far more costly, both economically and in community health, and therefore far more important to control than previously thought.

Every year there are about 2 billion cases of diarrhoea worldwide [6], a condition that is the second leading cause of mortality in children below the age of 5 years [7]. In recognition of the danger posed by diarrhoea, The United Nation’s Millennium Development Goal (MDG) number 4 aims to reduce childhood mortality by two-third by the year 2015 [8]. This review seeks to elucidate the contribution of diarrhoea to infant mortality, its impact on the realization of the MDG, and explores the possibilities of raising the chances of achieving this MDG.

## Methods

A PubMed search uses the keywords "acute infant diarrhoea” together with “prevalence”, “management”, or “prevention”; and “Millennium development goal 4” yielded six hundred and thirty (630) articles of which, twenty three (23) were found useful and seven (7) were found to be highly relevant to the review. Bibliographies of relevant studies and reviews were also included.

## Results

Results of the literature search are categorised under the following subtopics:

**Prevalence**

**Figure 1:**
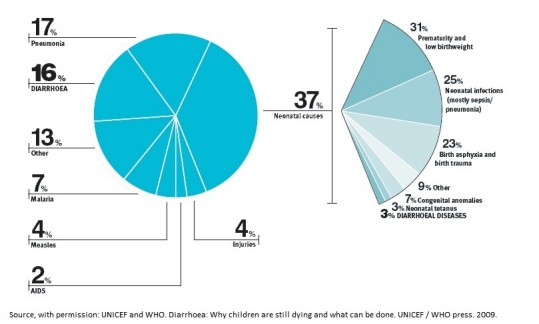
Proportional distrbution of cause-specific deaths among children under-five years of age. Reproduced with permission from: WHO. Diarrhoa: Why children are stil dying, and what can be done, 2009

For decades, diarrhoea has been described as one of the leading causes of mortality in the developing world, and also in the developed. The implications are however, more evident in the former [3]. In 1979, WHO reports [9] expressed concern on the matter and more than 2 decades later, the same agency reported diarrhoea as the second leading cause of infant mortality worldwide. Despite being an easily preventable and treatable disease, it causes 1.5 million deaths in children below the age of 5 years [7]. This follows closely behind pneumonia, the leading killer of young children. Together, pneumonia and diarrhoea account for an estimated 40 per cent of all global child deaths, each year, and the toll of diarrhoea alone is greater than that of AIDS, malaria and measles combined [7] (Figure 1).

**Figure 2:**
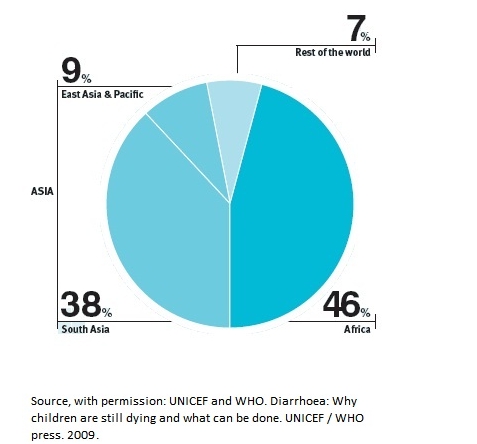
Proportional distribution of deaths due to diarrhoea diseases among children under five of age, by region. Reproduced with permission from: WHO. Diarrhoa: Why children are still dying, and what can be done, 2009

These figures may well be an underestimate excluding extreme rural cases that may not have been fortunate to make it to a medical facility. A WHO survey showed that 46% of the deaths occur in Africa alone (Figure 2). Furthermore, out of 15 countries with the highest infantile diarrhoea mortality worldwide, 10 are African countries, with Kenya ranking 10th [7] (Table 1).

**Management**

The foundation of management is fluid and electrolyte replacement and the enteric administration of food to prevent or correct a catabolic state and to promote enterocyte regeneration. The diarrhoea is usually self-limiting, and in mild cases, increased fluid administration combined with normal or reduced feeding is often sufficient to prevent dehydration. When the losses are greater due of numerous, watery stools and/or frequent vomiting, so that dehydration becomes clinically manifested, the patient is rehydrated with oral rehydration solution (ORS) and then given appropriate food.

Oral Rehydration Solution (ORS)

ORS was developed after the discovery of the sodium-glucose co-transporter pumps. Sodium is more effectively absorbed from the intestinal lumen by the SGLT-1 transporter together with glucose or galactose. Water then passively follows. ORS is hypo-osmolar and contains sodium and glucose in an optimum ratio for maximal uptake of sodium and water [2]. Thus, it should be administered in the prescribed dilution in water and not be mixed with beverages such as soft drinks.

Since the 1970s, ORS has been the cornerstone of management, to prevent life-threatening dehydration associated with diarrhoea. It has saved over 40 million children, since it was first tried during a cholera outbreak among Indian refugees during the Bangladesh war in 1971 [10]. The WHO task force of 2001 and the IAP National task force of 2003 both recommended that every doctor globally should prescribe ORS for all types of diarrhoea in all age groups [11]. In severe cases that may include shock, intravenous administration will prevent the progression to kidney failure and death [4].

It is worth noting that ORS is probably the cheapest intervention in medical practice, yet it remains grossly underused [10]. Studies have shown a decreasing trend in compliance, with Kenya ranking first [1]. This has been attributed to poor distribution, poor awareness and the fact that because it does not reduce faecal output it is perceived to be ineffective [10].

The standard WHO ORS initially contained a sodium and glucose concentration of 90 mEq/L each, which corresponds to the stool electrolyte composition in toxin-mediated diarrhoea [11]. Concerns were however raised on its hypernatremic effect and a low osmolarity ORS containing a concentration of 75 mEq/L was later developed. This is now officially recommended by WHO and UNICEF [1,11], and has also been found to be more effective [13]. The addition of bicarbonate and/or citrate has also been explored to facilitate rapid correction of metabolic acidosis [2].

In order to reduce the likelihood of vomiting, small quantities of the ORS should be regularly given with a teaspoon or a 5 mL syringe. If these portions are tolerated, the amount can be increased and the period between administrations lengthened. If the ORS is refused or vomited, its continuous administration through a nasogastric tube is significantly better than intravenous therapy with regard to the duration of diarrhoea, the length of hospital stay, and cost [2].

Zinc Supplementation

With the widespread Zinc deficiency among children [11], its supplementation has been recommended by WHO, UNICEF, and countries around the world for the treatment of diarrhoea [14]. The recommended dose is 20 mg for children and 10 mg for infants below the age of 6 months. [15]. Zinc has been shown to play a critical role in cellular growth and immune function that are vital for recovery [11]. It also provides substantial benefit in the reduction of stool output, reduced disease duration, faster weight gain following recovery, and thus improves prognosis [16,17]. Zinc supplementation therefore, lowers the cost of diarrhoeal management [18], hence the development of Zinc fortified ORS [11].

Anti-diarrhoeal drugs

These drugs include Loperamide, opiates, and anticholinergic mediators among others. Loperamide, for example, works through the opioid receptors of the enteric nervous system. It not only decreases intestinal motility, but reduces secretions as well [19], thus making it an effective antidiarrheal agent. Despite their efficacy, these drugs are not recommended for uses in infants due to their potential side effects. In addition, they prevent expulsion of pathogens, and possibly allow them to flourish.

Antibiotics

Antibiotics may be used in the treatment of some types of infant diarrhoea, specifically dysentery, where cotrimoxazole is the first line drug. In the event of resistance, nalidixic acid is recommended [11]. Anti-microbials are also obligatory for use in cases of infection with Salmonella, Vibrio cholera, Entamoeba histolytica, and Giardia lamblia [2].

Antiemetics

Vomiting is common in infant diarrhoea. Antiemetics are prescribed when the vomiting is severe, and low osmolarity ORS is not able to stop it. Concerns about their side effects have however been raised, and domeridone has been shown to be the safest since it has no side effects on the central nervous system [11]. The aim is not only to reduce the distress of vomiting, but also to lower water and electrolyte loss from the already dehydrated child. They are however not commonly recommended for use in infants, as they have been shown to increase diarrhoea [20].

Probiotics

Being non-virulent micro-organisms that when ingested, exert a positive influence on the health or physiology of the host; probiotics are occasionally used in the management of acute infant diarrhoea. Micro-organisms such as Lacto-bacillus acidophilus and Saccharomyces boulardii have been shown to reduce the duration of diarrhoea by enhancing mucosal immune responses and to compete with pathogens for attachment on the mucosa [11,21]. The mechanism of action is however, not known, and it is not commonly used [2].

Several combinations of the above management strategies may be co-administered for optimal results. ORS is however, the backbone of infant diarrhoeal management, and must always be included to ensure adequate rehydration. Low osmolarity ORS is more effective as it serves more purposes than rehydration alone. It reduces development of hypernatremia, hydrates more adequately and even has antiemetic properties.

Dietary recommendations

It is recommended that diarrhoeal infants resume normal feeding not more than four to six hours following the commencement of oral rehydration

[22]. This is due to the fact that enterocytes obtain nutrients mainly from the lumen, rather than from the blood, and will require sufficient supply for repair of the mucosa [23].

**Prevention**

Various preventive methods were reported in the literature, ranging from hygiene and diet, to drugs and supplements.

General hygiene

High hygiene standards must be maintained in order to minimise the transmission of pathogens especially during defecation, urination, and the handling of food [2].

Breastfeeding

Breast milk has immune functions. It contains IgA antibodies [24] that prevent attachment of pathogens to the gastrointestinal mucosa, thus preventing infections, including gastroenteritis [25].

Immunization

Immunization is one of the most effective ways to prevent disease. Considering the fact that rota virus is the commonest cause of infant diarrhoea, the development of a rota virus vaccine was a major milestone that saw a drastic reduction of infant diarrhoea death toll [26].

Zinc supplementation

Zinc supplementation is not only curative, but preventive too. It has been documented to reduce the incidence and prevalence of acute infant diarrhoea. It is believed to enhance immunity via the lymphoid lineage [11,27].

Probiotics

Probiotics too have been shown to be preventive. When administered to rotavirus diarrhoea infants, they have been shown to reduce the viral shedding, and hence reduce transmission [28].

## Discussion

From the above findings, it is interesting to note that despite the wide range of treatment and prevention modalities that are available, diarrhoea still remains a major contributor to infant mortality worldwide. This is an impediment to the achievement of Millennium Development Goal number 4.

The goal aims to reduce infant mortality by two-third by the year 2015. Recent studies, however, show minimal progress, and the MDG is, therefore, likely to be missed [29]. Despite the fact that the death toll has fallen from 4.5 million in 1979 to 1.6 million annual deaths [15], a lot still needs to be done to ensure the realization of the MDG.

It is of great concern, that diseases such as HIV/AIDS and malaria enjoy immense attention, with a great deal of research and campaign, yet the two afflictions cause only 2% and 7% infant deaths respectively [7]. Diarrhoea on the other hand, is becoming the forgotten killer despite its high mortality of 16% infant deaths. More effort needs to be made to educate the public on the available prevention and management modalities. Furthermore, research on diarrhoea and its cures needs to move away from the conventional treatments and explore the non-conventional. Focus needs to be placed on options like ethnopharmacological alternatives which will suit rural inhabitants better due to ready availability of herbs, and low cost of treatment. We took a leading role by conducting an in vitro study of the effect of organic extract of Mangifera indica on rabbit jejunum [30]. The study proved the potency of the extracts in treating diarrhoea, by demonstrating efficacy, and mode of action [30]. Due to the widespread growth of the plant, it is felt that it can make a major impact in the treatment of infant diarrhoea.

Non-conventional techniques should also be explored, an example being faecal biotherapy. This technique of transplanting processed faecal matter from a healthy individual to a diseased one in order to restore colonic normal flora has faced immense social criticism. It has as a result not made significant advancement ever since it was first described in 1958 [31]. The human faecal flora is a complex mix of organisms, containing almost nine times more living cells than does the entire body [32]. Faecal biotherapy has proven to be an effective cure for Pseudomembranous colitis diarrhoea caused by Clostridium dificile [31,33] with more than 95% success rates [34]. This form of diarrhoea is an excruciating type that can cause more than 40 bowel movements, per day. It is commonly fatal if left uncontrolled [35].

Faecal transplant has been shown to cause a dramatic recovery and patients occasionally require only one session of treatment, as the introduced normal flora rapidly displaces the attached pathogenic Clostridium dificile, hence restoring normal faecal physiology. It is believed that it has the potential to treat other colonic disorders. Furthermore, it is likely to prove extremely cost effective due to its minimal requirements, and may in our view be the ignored answer to attainment of the MDG.

## Conclusion

It is clear that diarrhoea is a major obstacle in the realization of MDG-4. Ironically however, it is a disease that is so easily treatable and preventable that it should not cause such high mortality rates. A review of existing strategies and formulation of newer ones is, therefore, necessary. Research priorities need to be refocused from what are perceived as killer diseases by the developed world, because far more children die of diarrhoea in one day than have ever died from avian influenza for example.

## Acknowledgments

Professor Charles OA Omwandho, Associate Professor of Biochemistry, Dean of the School of Medicine, University of Nairobi.

## Competing interests

The authors declare no competing interests.

## Authors’ contributions

All authors contributed equally towards this manuscript.

## Figures and Tables

**>Table 1:** 

Rank	Country	Total number of annual child deaths due to diarrhoea
1	India	386,600
2	Nigeria	151,700
3	Democratic Republic of the Congo	89,900
4	Afghanistan	82,100
5	Ethiopia	73,700
6	Pakistan	53,300
7	Bangladesh	50,800
8	China	40,000
9	Uganda	29,300
10	Kenya	27,400
11	Niger	26,400
12	Burkina Faso	24,300
13	United Republic of Tanzania	23,900
14	Mali	20,900
15	Angola	19,700

Source, with permission: UNICEF and WHO. Diarrhoea: Why children are still dying and what can be done. UNICEF / WHO press. 2009.
